# Changes in cerebral glucose metabolism after 3 weeks of noninvasive electrical stimulation of mild cognitive impairment patients

**DOI:** 10.1186/s13195-016-0218-6

**Published:** 2016-12-01

**Authors:** Kyongsik Yun, In-Uk Song, Yong-An Chung

**Affiliations:** 1 Computation and Neural Systems, California Institute of Technology, 1200 East California Boulevard, Pasadena, CA 91125 USA; 2 Department of Neurology, Incheon St. Mary’s Hospital, The Catholic University of Korea, #56 Dongsu-ro, Bupyeong-gu, Incheon, 21431 South Korea; 3 Department of Radiology, Incheon St. Mary’s Hospital, The Catholic University of Korea, #56 Dongsu-ro, Bupyeong-gu, Incheon, 21431 South Korea

**Keywords:** Mild cognitive impairment, Transcranial direct current stimulation, Positron emission tomography

## Abstract

**Background:**

Mild cognitive impairment (MCI) is a syndrome that disrupts an individual’s cognitive function but preserves activities of daily living. MCI is thought to be a prodromal stage of dementia, which disrupts patients’ daily lives and causes severe cognitive dysfunction. Although extensive clinical trials have attempted to slow or stop the MCI to dementia conversion, the results have been largely unsuccessful. The purpose of this study was to determine whether noninvasive electrical stimulation of MCI changes glucose metabolism.

**Methods:**

Sixteen MCI patients participated in this study. We used transcranial direct current stimulation (tDCS) (2 mA/day, three times per week for 3 weeks) and assessed positron emission tomography (18 F-FDG) before and after 3 weeks of stimulation.

**Results:**

We showed that regular and relatively long-term use of tDCS significantly increased regional cerebral metabolism in MCI patients. Furthermore, subjective memory satisfaction and improvement of the memory strategies of participants were observed only in the real tDCS group after 3 weeks of stimulation.

**Conclusion:**

Our findings suggest that neurophysiological intervention of MCI could improve glucose metabolism and transient memory function in MCI patients.

## Background

Mild cognitive impairment (MCI) is a syndrome that impairs an individual’s everyday cognitive function more than expected for their age and education level but does not affect the activities of daily life. Although MCI is distinct from dementia, which disrupts patients’ daily functions and produces more severe cognitive deficits, MCI patients with memory impairment have been shown to have a high risk of progression to dementia [1]. It is estimated that 3–19 % of the general population of individuals older than 65 years exhibit MCI, and 11–33 % of these individuals have a risk of progressing to dementia within 2 years [2]. More than half of MCI patients with both vascular disease and cognitive impairment progress to dementia within 5 years [3]. Thus, MCI can be thought of as a prodromal phase of dementia, and early diagnosis and intervention have great potential to prevent cognitive decline that is severe enough to interfere with patients’ daily lives.

Many clinical attempts have been made to prevent the progression of MCI to dementia, but the results have largely been unsuccessful [4]. Numerous major clinical trials have used acetylcholinesterase inhibitors (AChEIs) for MCI treatment [5] because MCI patients are thought to have a central cholinergic deficit and loss of nucleus basalis neurons [6]. Galantamine, an AChEI, has been tested for the treatment of MCI, but there was no significant difference in the progression rate to dementia between the galantamine and the placebo groups [7, 8]. There was also no significant difference in the progression rate from amnestic MCI to dementia in patients treated with vitamin E or donepezil [9]. Amnestic MCI patients treated for 24 weeks with donepezil showed no improvement in the performance of a delayed recall memory test [10]. Based on these clinical results, a Cochrane review concluded that no evidence exists to indicate that donepezil is beneficial for MCI patients [11]. Although several trials are underway to determine whether antioxidants or cognitive stimulants might slow the MCI to dementia conversion [9], the results so far have been disappointing; only one randomized trial of a small group treated with a dopamine agonist showed a significant improvement in the Mini-Mental State Examination score (MMSE) [12].

These clinical outcomes failed to provide proof that drugs used for treating MCI can precisely target the neurophysiological objectives. Recently, transcranial direct current stimulation (tDCS), a method used to noninvasively stimulate specific cortical regions of the brain with a mild (<2 mA) and persistent current [13–15], has shown a clinically significant effect on various neuropsychiatric diseases [16]. In patients with depression, 1–2 weeks of treatment with tDCS improved both the symptoms and psychological scale of patients on the Montgomery-Asberg Depression Scale (MADRS) [17, 18] even more than conventional psychiatric drugs that selectively block the serotonin transporter [19].

In dementia, a single use of tDCS improved recognition memory [20] and visual recognition memory [17], and these effects were also observed after 1 month if tDCS was applied in daily sessions for 5 days [21]. Similarly, a study of MCI patients showed that tDCS application improved recall performance, but this relationship was not observed in the sham condition [22]. These results suggest that neurophysiological intervention in the early stage of cognitive impairment could improve the neuropsychological performance of MCI and dementia patients [23]. The electrical stimulation targets the prefrontal cortex which governs the various cognitive functions, including working memory, visual recognition, executive attention, and general fluid intelligence [24, 25]. Therefore, we may relate the cognitive improvement to the increase of neuronal activity of the prefrontal cortex induced by tDCS [26, 27].

Electrical stimulation of the brain has been known to produce brain-derived neurotrophic factor (BDNF) that increases synaptogenesis and neurogenesis in the long term [28–30]. Therefore, tDCS may induce synaptic plasticity and neuronal viability [30]. We hypothesize that these effects may benefit the treatment of MCI.

Although previous studies have attempted to measure the behavioral outcome of MCI patients after tDCS treatment, it is largely unknown how cerebral function and metabolism are altered by the use of tDCS. We hypothesize that the cerebral glucose metabolism would be changed after tDCS, because it has been known that the neuronal activity induced by electrical stimulation increases glucose metabolism [31–33]. Furthermore, previous studies have largely depended on single or short-term (less than 1 week) sessions to measure the effects of tDCS on MCI and dementia. Thus, we aimed to investigate how regular and relatively long-term (3 weeks) treatment with tDCS might affect cerebral metabolism and enhance the cognitive performance of MCI patients. To measure cognitive changes, we used the Multifactorial Memory Questionnaire (MMQ), a reliable and valid method of quantifying the effect of treatment over the span of several weeks [34, 35].

## Methods

### Participants

Sixteen patients with MCI, aged 65–85 years, participated in this study. They were randomly distributed between the active and the sham groups. The diagnosis of MCI was consistent based on the following criteria proposed by Petersen et al. [36]: memory complaints, normal activity of daily living, normal general cognitive function, abnormal memory for age, and lack of dementia. All subjects were evaluated in the dementia clinic by an experienced neurologist and psychologist. The evaluation procedure consisted of a detailed medical history, physical and neurologic examinations, neuropsychological assessments, and brain magnetic resonance imaging. Additionally, positron emission tomography using 18 F-fluoro-2-deoxyglucose (FDG-PET) was also performed on all subjects. The patients were selected independent of FDG-PET hypometabolism. The patients’ past medical histories were obtained from the patients and family members or from other caregivers. The MCI patients had never had parkinsonian symptoms or the focal neurological signs or radiological lesions that typify cerebrovascular diseases. Additionally, MCI patients were excluded if they had psychiatric disorders, mental retardation, drug intoxication, or diabetes mellitus. All participants provided written informed consent after receiving a detailed explanation of the experimental procedures. The Institutional Review Board of the Catholic University of Korea approved all experimental procedures for this study.

### Neuropsychological testing

Patients’ general cognitive state and severity of dementia were evaluated using the MMSE [37, 38], the extended version of the Clinical Dementia Rating (CDR) [39], and the sum of the box score of the CDR (SOB) [40]. Several cognitive domains were assessed by a detailed neuropsychological battery of tests, including an attention test (forward digit span, backward digit span, and calculation), a language and related function test (Boston Naming Test), a visuospatial function test (the Rey Complex Figure Test (RCFT)), a verbal memory test (three-word registration and recall, Hopkins Verbal Learning Test (HVLT) for immediate recall, delayed recall, and recognition), a nonverbal memory test (immediate recall, delayed recall, and recognition of a Rey complex figure), and a frontal executive function test (controlled oral word association test (animal, supermarket, and letter)) [41].

To assess the cognitive function and subjective memory complaints of participants, we also used the modified MMQ [42]. This questionnaire measures the self-appraisal of memory function and consists of 57 items classified into three subscales including MMQ-C (contentment with current memory function), MMQ-A (self-appraisal of current memory ability in daily life), and MMQ-S (use of everyday memory strategies and aids). Before each experiment, participants reported their memory ability, including overall contentment or satisfaction with their own memory ability.

### Procedure

#### Protocol for tDCS sessions

In this randomized, double-blind study, patients received nine active or sham tDCS sessions (three times per week for 3 weeks). In the active tDCS condition, stimulation was administered at 2 mA for 30 minutes, and the current was gradually ramped up over 20 seconds. In the sham tDCS condition, to provide participants with the same initial sensation of tDCS, the amplitude, duration, and locations of the anodal and cathodal tDCS were identical, but the current was gradually ramped down after the first 20 seconds. We followed the stimulation protocols of the previous studies using tDCS [32, 33]. The randomized, double-blind assignment was performed using a simple random number generator from the Matlab software by a laboratory researcher independent of the experiment. This researcher put the randomly generated number into the tDCS device to randomly select between the real and sham trials. The experimenter was not informed whether the current trial is the real or the sham condition. The patients were stimulated with a DC stimulator developed by Yun and colleagues [15]. The anodal electrode was placed on the left dorsolateral prefrontal cortex (DLPFC) (F3; 10–20 EEG system), and the cathode electrode was placed on the right DLPFC (F4; 10–20 EEG system). The regions were selected based on the previous tDCS studies for cognitive improvement [43–45]. The conductive rubber electrodes used for tDCS were potassium chloride-soaked sponges (5 cm × 5 cm = 25 cm^2^). They were held in place by a headband. The patients were asked to report any adverse effects, including itching, tingling, headache, burning sensation, and discomfort [46]. No patient reported adverse effects.

**Fig. 1:**
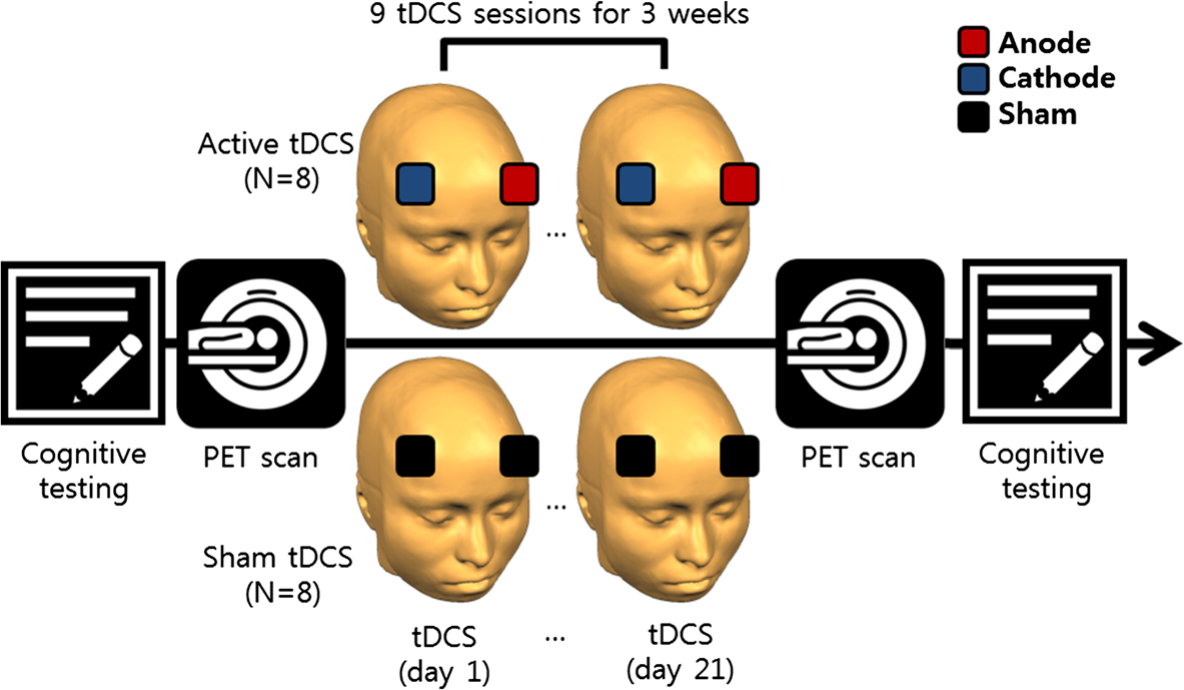
Experimental procedure. Participants received nine active or sham tDCS sessions (three times per week for 3 weeks). On the first day of the experiment, the participants performed a set of cognitive tests and PET imaging. The participants then moved to the adjacent testing room for the tDCS session. From the second to the eighth visits, the participants performed only the tDCS sessions. On the last day of the experiment (ninth visit), the participants performed the tDCS session, PET scan, and cognitive tests consecutively. *PET* positron emission tomography, *tDCS* transcranial direct current stimulation

On the first day of the experiment, the participants performed a set of neuropsychological tests and they moved to the PET room to obtain functional brain images of glucose metabolism. Then the participants moved to the adjacent testing room for the tDCS session. After the first day of the experiment, the participants were asked to schedule eight more hospital visits for the next 3 weeks (three visits per week from Monday to Friday). From the second to the eighth visits, the participants performed only the tDCS sessions. On the last day of the experiment (ninth visit), the participants performed the tDCS session, PET scan, and cognitive testing consecutively (Fig. 1).

FDG-PET and the modified MMQ [42] were used to assess the cognitive ability of participants at baseline and after 3 weeks of treatment. After the sessions, FDG-PET images were acquired to measure the metabolic activity of the participants.

#### PET hardware and imaging

FDG-PET brain scans were performed on the Discovery STE PET/CT scanner (GE Healthcare, Milwaukee, WI, USA) using standard techniques. Forty-seven horizontal slices were acquired with 3.27 and 3.75 mm transverse resolution and 1.95 and 0.488 mm resolution. All scans were performed as the participants rested with their eyes closed in a quiet, dimly lit room. The participants were required to fast for at least 4 hours. The measures of regional cerebral glucose metabolism were obtained following the administration of a 185–222 MBq intravenous injection (2-minute period) of FDG. The emission scans were obtained after a 45-minute uptake period. The images from 45–65 minutes post injection were used for the analysis. The whole brain glucose metabolism was obtained using standard filtering and reconstructing techniques.

### Data analysis

#### Behavioral data analysis

Independent *t* tests were performed to confirm that there were no differences in age, education, MMSE, CDR, and HVLT scores between the active tDCS and the sham groups. Nonparametric chi-square test was performed to confirm that the gender distribution between the two groups was not significantly different. MMQ scales before and after the tDCS treatments were compared using paired *t* test. The significance level was set at *p* < 0.05.

#### PET imaging analysis

The PET scans were interpolated into 47 slices, corrected for slice acquisition time within each volume, motion corrected with realignment to the first volume, registered, transformed into the coordinates of the MNI standard space (Montreal Neurological Institute, McGill University, Montreal, Canada), and smoothed to 8 mm in the *x*, *y*, and *z* planes using statistical parametric mapping (SPM 8; Wellcome Department of Imaging Neuroscience, Institute of Neurology, London, UK).

Two comparisons were performed between baseline and follow-up active groups and between active and sham groups after 3-week tDCS treatment. Covariates were not considered in this analysis because of the small sample size. We computed the post-hoc analyses for the regional PET values using *p* < 0.05, corrected by a false discovery rate (FDR) for multiple tests for baseline and after treatment comparisons of the active group and for active and sham comparisons after 3-week treatment. The interaction between time (pre and post stimulation) and groups (real and sham) was also calculated.

## Results

In this study, 16 patients with MCI received active or sham tDCS sessions over 3 weeks. The clinical characteristics of all participants are summarized in Table 1. Between the two groups, there were no significant differences in age (statistical values, *t*(14) = 0.534; *p* = 0.602), MMSE scores (statistical values, *t*(14) = 1.450; *p* = 0.169), delayed recall with the HVLT (statistical values, *t*(14) = 0.752; *p* = 0.465), RCFT scores (statistical values, *t*(14) = 0.883; *p* = 0.392), or S-IDAL scores (statistical values, *t*(14) = –0.395; *p* = 0.699).

**Table 1:** Clinical characteristics during the baseline assessment

	Real tDCS group, mean (SD)	Sham tDCS group, mean (SD)	*p* value	Mann–Whitney *U* test
Age (years)	74.75 (7.47)	73.12 (4.25)	0.60	*U* = 30.50, *p* = 0.87
Education (years)	8.06 (4.93)	5.56 (2.41)	0.22	*U* = 19.50, *p* = 0.19
Mini-Mental State Examination	26.75 (1.58)	25.12 (2.74)	0.16	*U* = 18.50, *p* = 0.16
Clinical Dementia Scale	0.25 (0.26)	0.50 (0.26)	0.08	*U* = 18.00, *p* = 0.16
Gender (male/female)	3/5	2/6	χ^2^ = 2.25 *p* = 0.13	χ^2^ = 2.25, *p* = 0.13
Hopkins Verbal Learning Test	4.37 (3.42)	3.12 (3.22)	0.46	*U* = 25.00, *p* = 0.50

*tDCS* transcranial direct current stimulation

### Behavioral results

**Fig. 2:**
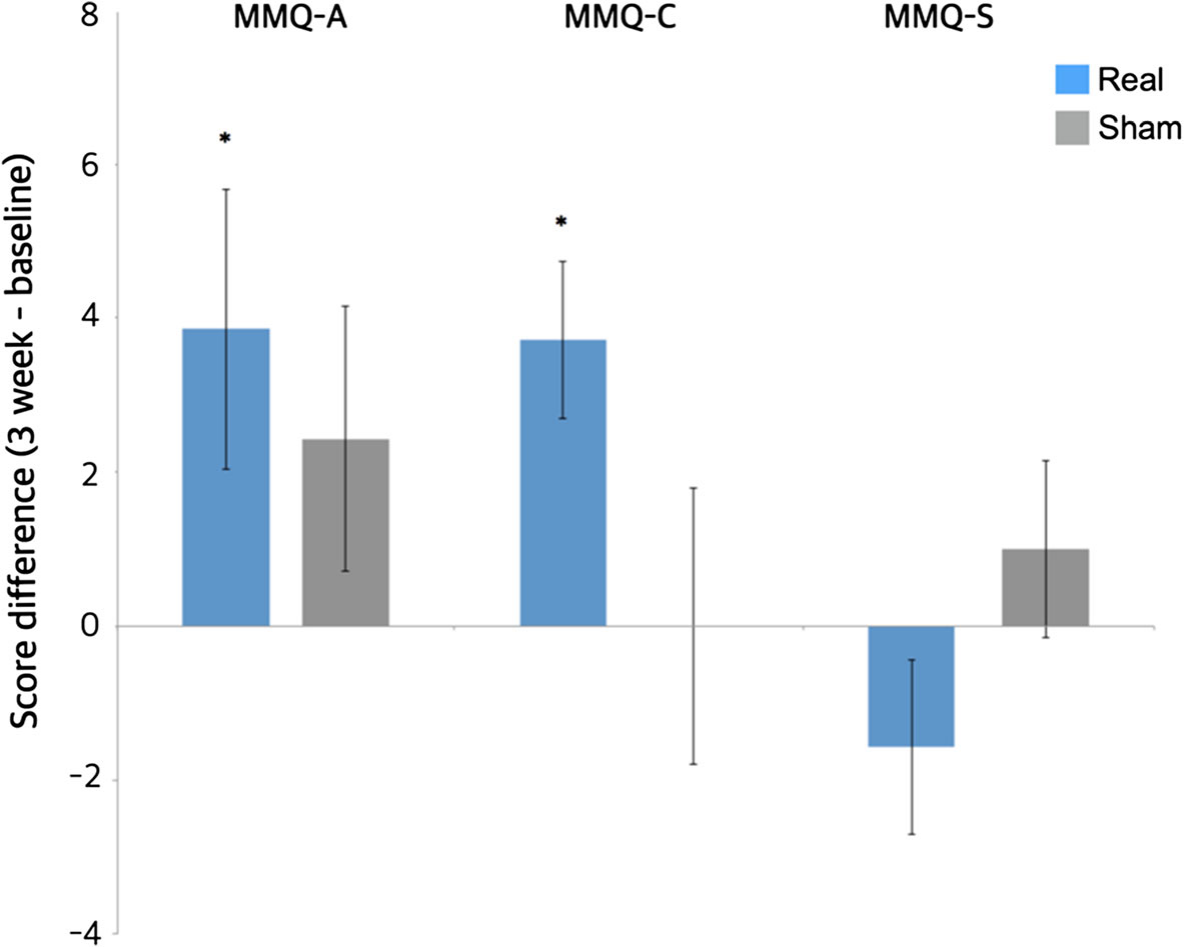
Participants’ memory complaints measured by the MMQ scale. The MMQ-A subscale assesses memory lapses in everyday activities such as names of people and locations of items. MMQ-A was improved significantly after 3-week tDCS application only in the real tDCS group (paired *t* test, *p < 0.05). The MMQ-C subscale measures the subjective satisfaction of patients’ memory abilities and determines whether subjects experience anxiety regarding their memory problems. MMQ-C was also significantly improved after 3-week tDCS application only in the real tDCS group (paired *t* test, *p < 0.05). There was no significant difference between real and sham groups in the MMQ-S subscale, which measures the subject’s memory strategies for compensation. *Error bar* denotes standard deviation. *MMQ* Multifactorial Memory Questionnaire

Participants’ memory complaints were measured using the MMQ scale and are presented in Fig. 2. MMQ scores consist of MMQ-A (ability), MMQ-C (contentment), and MMQ-S (strategy). The interaction between the test scores (score differences between post and pre stimulation) and the group (real and sham) showed that the real and the sham tDCS effects in the test scores were significant (*F*(2) = 4.13; *p* = 0.05). In the real tDCS stimulation group, the MMQ-C score was significantly increased after 3 weeks of tDCS stimulation (*t*(15) = 2.15; *p* = 0.048, two-tailed), but there was no significant difference in the sham condition group (*t*(15) = 1.91; *p* = 0.09, two-tailed). Similar results were observed for the MMQ-A scale, with a significant improvement in the tDCS stimulation group after 3 weeks of stimulation (*t*(15) = 3.65; *p* = 0.002, two-tailed). However, the subjective scale of the MMQ-A was not significantly different in the sham tDCS group (*t*(15) = 0.23; *p* = 0.82, two-tailed). The MMQ-S subscale, which measures patients’ everyday memory strategies and aids, was not significantly different in both the real tDCS and sham stimulation groups (real tDCS group: *t*(15) = 1.39; *p* = 0.21 and sham tDCS group: *t*(15) = 0.58; *p* = 0.57, both two-tailed).

### Changes in metabolic rates

**Fig. 3:**
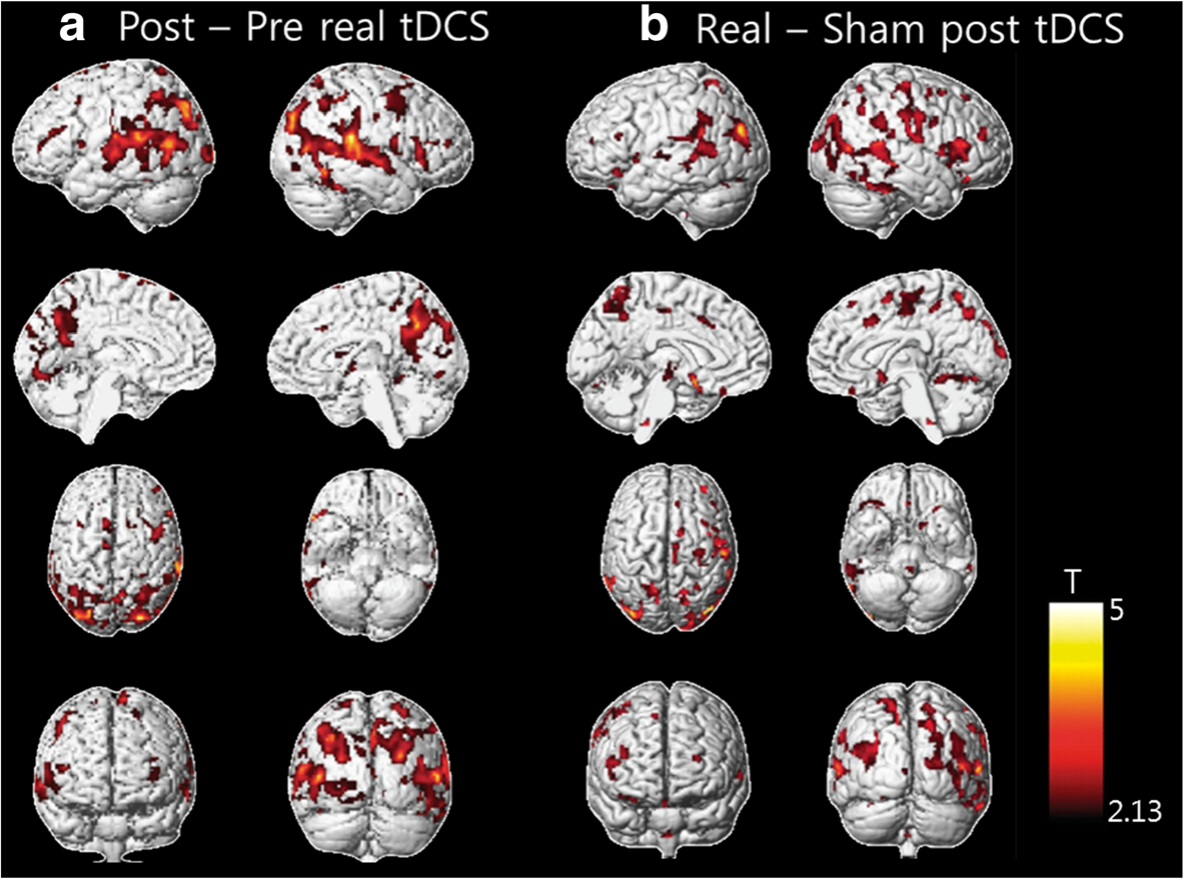
PET results after 3 weeks tDCS > baseline and tDCS > sham. **a** Brain regions showing increased glucose metabolism after 3-week tDCS treatment versus baseline condition. **b** Brain regions showing significantly increased glucose metabolism in the real tDCS stimulation group versus the sham group (*p* < 0.05, FDR corrected). *tDCS* transcranial direct current stimulation

After 3 weeks of active tDCS treatment, increased metabolism was observed in the dorsolateral, ventrolateral, and medial prefrontal cortices, the dorsal anterior cingulate, the anterior and posterior insular regions, and the hippocampal and parahippocampal regions (Fig. 3a; *p* < 0.05, FDR corrected) (Table 2). This result suggests that multiple tDCS treatments significantly increase individual brain metabolic activity during resting conditions.

**Table 2:** Contrast between post-treatment and pre-treatment conditions in the active transcranial direct current stimulation group

Region	*x*	*y*	*z*	*t* value
Dorsolateral prefrontal	40	34	29	4.40
Ventrolateral prefrontal	–54	24	3	3.50
Medial prefrontal	–9	39	–6	3.41
Dorsal anterior cingulate	10	6	46	2.36
Anterior insula	37	20	0	3.36
Posterior insula	–39	1	9	2.49
Hippocampal	–29	–16	–21	3.67
Parahippocampal	18	–17	–16	3.82

To assess whether these increased activities after tDCS treatment were derived from a placebo effect, we compared the active tDCS treatment group with the sham control group. After treatment, the active group demonstrated significantly higher metabolic activity in the medial prefrontal cortex, the precuneus, the midtemporal regions, and the anterior cingulate cortices than the sham group (Fig. 3b; *p* < 0.05, FDR corrected) (Table 3). This result suggests that increased brain metabolism after tDCS treatment might be related to the direct current stimulation rather than the placebo effect. We did not find any significant regions in the interaction analysis between time (pre and post stimulation) and groups (real and sham). This result may be due to the small sample size.

**Table 3:** Contrast between the active and the sham tDCS groups post treatment

Region	*x*	*y*	*z*	*t* value
Medial prefrontal	12	64	5	3.55
Precuneus	–10	–52	68	2.38
Midtemporal	–50	–40	–2	2.40
Anterior cingulate	0	33	6	3.33

## Discussion

In this study we observed the longitudinal effects of tDCS in MCI patients and measured metabolic activity in pre-treatment and post-treatment conditions. In the active tDCS group, brain metabolism was significantly increased after 3 weeks (total nine sessions) of tDCS treatment, and the post-treatment brain metabolism was significantly higher in the active tDCS group than in the sham group. Previous studies have shown that even a single session could enhance the cognitive performance in dementia patients [16, 17, 20]. Our findings suggest that regular and frequent administration of tDCS in MCI patients can modulate the metabolism of certain brain regions as well as enhance neuropsychological performance. Improvement of cognitive function after tDCS might not be a transient effect, because regular stimulation (i.e., daily sessions for 5 days) improved visual recognition memory for 4 weeks after stimulation [21].

We found that the cerebral metabolic activity of MCI patients significantly increased after tDCS administration, especially in the active treatment group compared with the sham control group. A previous study showed that MCI and dementia patients have higher 2-(1-(6-((2-fluoroethyl) (methyl) amino)-2-naphthyl)ethylidene) alononitrile (FDDNP) binding and lower 2-deoxy-2-[F-18]fluoro-D-glucose (FDG) uptake in the temporal, parietal, posterior cingulate, and frontal regions [47]. Two proteins, beta-amyloid and tau, are abnormally accumulated in these regions in dementia patients [48], and neurofibrillary tangles have also been detected in the medial temporal and hippocampal regions in MCI patients [49]. This neuropathological finding might correlate with metabolic activity of the brain, because the patients with dementia showed significantly lower glucose metabolism in similar regions, such as the parietal, temporal, frontal, and posterior cingulate cortices [50]. In this study, we found increased FDG uptake in multiple brain regions, including the anterior and posterior insular, hippocampal, and parahippocampal regions, especially in the active tDCS administration group. Previous studies showed that tDCS increased the brain metabolism as compared with sham stimulation [51, 52]. Therefore, we speculate that increased brain metabolism and cognitive improvements in MCI patients were due to a noninvasive neuromodulatory effect rather than a placebo effect.

Furthermore, we found that participants’ reports of their memory ability, which was measured by the MMQ-A and MMQ-C subscales, were significantly improved only in the real tDCS group. The MMQ-A subscale consisted of 20 items and assessed memory lapses in everyday activities such as names of people and locations of items [53]. A higher score on the MMQ-A subscale indicates individuals who are less likely to experience memory problems in their daily life and are satisfied with their own memory function. The MMQ-C subscale also measures the subjective satisfaction of patients’ memory abilities and determines whether subjects experience anxiety regarding their memory problems. Although participants in our study did not know whether they were assigned to the real or sham tDCS group, both the MMQ-A and MMQ-C scores were significantly higher only in the real tDCS group after 3 weeks of stimulation. This result suggests that regular use of tDCS might improve the overall contentment or satisfaction of patients with their memory ability as well as enhance their metabolic activity.

Previous studies have noted that MCI patients have a risk of progression to dementia, and the baseline memory performance significantly predicted the conversion to dementia [1]. Although various clinical interventions have examined symptomatic drug treatment for MCI patients, there is no significant evidence that anti-dementia drugs lower the progression rate from MCI to dementia during 1–3 years of treatment [4]. Regarding the therapeutic effect of tDCS on various neuropsychiatric diseases, such as depression, schizophrenia, and dementia [16], our findings suggest that regular and relatively long-term administration of tDCS might enhance cognitive performance in MCI patients.

One limitation of our study is the short observation period compared with conventional pharmacological interventions, which have a 6-month to 3-year treatment period [4]. Although the effect of tDCS is known to last for weeks after administration [21], a longer observation period might be needed to confirm whether the use of tDCS slows or stops the conversion of MCI to dementia. Furthermore, a recent study used more frequent tDCS administration consisting of daily sessions for 5 days during 1 week [21]. Although most studies have used a single session per subject [17, 20], the current tDCS protocol for MCI and dementia has not been optimized. Other studies have shown improvement in memory function of dementia patients after tDCS treatment [16], and the standardization of the duration, electrode size, and current strength of tDCS administration on MCI and dementia patients is needed. It should also be mentioned that the PET images and the test scores were the results of the combined effect of the long-term (3 weeks) and the short-term tDCS treatments because the last PET imaging and the neuropsychological testing were performed right after the last tDCS treatment. The experimental design was not optimal to exclude the acute effect of stimulation but we believe that the acute effect was still part of the long-term effect. Further long-term and large-scale research is warranted to confirm the effect of tDCS on MCI.

## Conclusion

We cautiously suggest through this study that neurophysiological intervention of MCI could improve transient memory function in MCI patients, although the study covers a short time period and involves a small number of subjects. Therefore, we believe that larger prospective studies investigating the clinical efficacy of neurophysiological intervention of MCI with a long-term follow-up period should be performed in the future to clarify whether neurophysiological intervention plays an important role in the improvement of memory function in MCI.

## Abbreviations

AChEI: Acetylcholinesterase inhibitor
CDR: Clinical Dementia Rating
DLPFC: Dorsolateral prefrontal cortex
FDDNP: 2-(1-(6-((2-Fluoroethyl) (methyl) amino)-2-naphthyl)ethylidene) alononitrile
FDG-PET: Positron emission tomography using 18 F-fluoro-2-deoxyglucose
FDR: False discovery rate
HVLT: Hopkins Verbal Learning Test
MCI: Mild cognitive impairment
MMQ: Multifactorial Memory Questionnaire
MMSE: Mini-Mental State Examination
RCFT: Rey Complex Figure Test
SOB: Sum of the box score of the CDR
SPM: Statistical parametric mapping
tDCS: Transcranial direct current stimulation
